# Characterizing the growth of PHA-producing microorganisms on short-chain carboxylic acids

**DOI:** 10.1186/s12934-025-02840-8

**Published:** 2025-09-23

**Authors:** Steven Leonhardt, Pravesh Tamang, Günter E. M. Tovar, Susanne Zibek

**Affiliations:** 1 Fraunhofer Institute for Interfacial Engineering and Biotechnology IGB, Stuttgart, Germany; 2 Institute of Interfacial Process Engineering and Plasma Technology IGVP, University of Stuttgart, Stuttgart, Germany

**Keywords:** Carboxylic acids, Polyhydroxyalkanoate, *Cupriacidus necator* H16, *Pseudomonas Putida* KT2440, *Azohydromonas australica* DSM 1124, And *Haloferax mediterranei* DSM 1411, Inhibition, Specific growth rate

## Abstract

Short-chain carboxylic acids are important chemical intermediates in anaerobic treatment or thermal hydrolysis of biowaste. They are a crucial precursor for the production of Polyhydroxyalkanoate (PHA) by various microorganisms. However, there is limited data on the growth behavior of PHA-producing microorganisms on carboxylic acids and their tolerance range. Therefore, this study aims to systematically determine the growth behavior of four commonly used PHA-producing microorganisms (*Cupriacidus necator*, *Pseudomonas putida*, *Azohydromonas australica*, and *Haloferax mediterranei*) on different carboxylic acids (formic, acetic, propionic, valeric, and levulinic acid). Batch experiments were conducted in a microbioreactor to determine the maximum specific growth rates (*µ*_*max*_) on varying acid concentrations. Additionally, the data was analyzed using the Han-Levenspiel growth model to determine the inhibitory effect of the acids on the microorganisms. Our experiments showed that *P. putida* had the highest *µ*_*max*_ on propionic acid (0.165 h^− 1^) and levulinic acid (0.16 h^− 1^). Likewise, *H. mediterranei* also showed the highest *µ*_*max*_ on propionic acid (0.174 h^− 1^). For *C. necator*, the highest *µ*_*max*_ was determined in acetic (0.102 h^− 1^) and levulinic acid (0.109 h^− 1^). Among all the tested carboxylic acids, acetic acid was found to be the least toxic acid and was also the only substrate that enabled meaningful growth of *A. australica*. Furthermore, formic acid seems to be the least suitable substrate for the growth of these microorganisms except *H. mediterranei* as only the growth of this microorganism was observed. Based on these results, we concluded that carboxylic acids are not a suitable carbon source for the growth of *A. australica.* While *C. necator* and *P. putida* are more versatile in utilizing various acids, except formic acid. They showed the largest optimum growth or tolerance range in acetic and levulinic acid, making these acids the most preferred substrate for growth. Lastly, *H. mediterranei* could grow in all carboxylic acids, however, the requirement of a highly saline medium would be challenging.

**Supplementary Information:**

The online version contains supplementary material available at 10.1186/s12934-025-02840-8.

## Introduction

Among the many marvels of microbial metabolite, polyhydroxyalkanoates (PHAs) have emerged as a promising alternative to conventional plastics. These biodegradable polymers are produced by a variety of microorganisms, including bacteria and archaea, in response to environmental stress and nutrient limitations. The significance of PHAs extends beyond their biodegradability; they also embody the principles of a circular economy as they can be produced from renewable feedstocks, including organic waste materials [1].

One particularly promising class of feedstocks for PHA production is short-chain carboxylic acids with 1–5 carbon atoms. These organic compounds are not only cost-effective but also highly sustainable, as they can be derived from various biowaste conversion processes such as anaerobic digestion and thermal hydrolysis [2–4]. Their direct role as precursors for poly-3-hydroxybutyrate (PHB) and poly-3-hydroxyvalerate (PHV) makes them particularly attractive for synthesizing the poly-(3-hydroxybutyrate-co-3-hydroxyvalerate), PHBV copolymer, which has enhanced material properties compared to PHB homopolymer [5, 6].

However, despite their potential, carboxylic acids present a significant challenge: toxicity to microbial cells. Depending on their concentration, extracellular pH, and hydrophobicity, these acids can diffuse into the cytoplasm, disrupt intracellular pH balance and inhibiting ATP generation [7]. These in turn could impose a critical limitation on biomass growth and PHA production, necessitating systematic research into microbial tolerance levels and optimized feeding concentration. Furthermore, Tao et al. where able to produce 53 g·L^− 1^ on a mixture of acetic and butyric using the bacterium *Salinivibrio spp*. This bacterium showed high tolerance to acetic acid of up to 100 acetate g·L^− 1^ [11].

Although cultivations on some carboxylic acids have been reported for several PHA producers like *Pseudomonas putida*, *Haloferax mediterranei*,* Salinivibrio spp.* and *Cupriavidus necator* [8–11], which are among the most commonly utilized microorganisms for PHA production, a systematic characterization of growth kinetic data is lacking for these bacteria.

Microorganisms such as *Azohydromonas australica* and *Haloferax mediterranei* offer unique advantages in the PHA production process, but they remain relatively underexplored. *A. australica* is known to produce PHA without the need for external stress factors, such as nitrogen or phosphate limitation, which are typically required by *C. necator* and *P. putida*. This distinctive metabolic trait, associated with an impaired TCA cycle which leads to an overflow metabolism of acetyl-CoA [12], has primarily been studied in sugar-based media. However, there is limited research on its growth in carboxylic acids, especially in the absence of co-substrates like sugars [13]. Meanwhile, the halophilic archaeon *H. mediterranei* presents a unique opportunity in biopolymer production. Unlike most microorganisms, it can synthesize PHBV even from non-related carbon sources, accumulating up to 10% 3-Hydroxyvalerate when grown on sugars [14]. Its remarkable tolerance to high salinity (up to 200 g·L^− 1^ NaCl) offers practical advantages, including reduced sterility requirements and the possibility of using seawater for cultivation. Additionally, its PHA extraction process is simplified, as osmotic pressure differences enable cell lysis just using fresh water.

This study aims to fill critical knowledge gaps by systematically investigating and comparing the growth kinetics of *Pseudomonas putida* KT2440, *Cupriavidus necator* H16, *Azohydromonas australica* DSM 1124, and *Haloferax mediterranei* DSM 1411 on various short chain carboxylic acids, including formic, acetic, propionic, butyric, and valeric acids, as well as levulinic acid. By analyzing inhibitory concentrations, lag phase variations, and species-specific tolerances, we seek to provide kinetic parameters for optimizing fermentation processes. Understanding these dynamics will pave the way for more efficient and scalable PHA production, bringing us one step closer to sustainable, bio-based materials that align with the principles of a circular economy.

## Materials and methods

### Microbial strains

The microorganisms *Cupriavidus necator H16*, *Pseudomonas putida KT2440*, *Azohydromonas australica DSM 1124* and *Haloferax mediterranei DSM 1411* used in this study have been purchased from the German Collection of Microorganisms and Cell Cultures (DSMZ). The strains were stored at -80 °C on 16.5% glycerol. For cultivation, the microorganisms were streaked on agar plates containing their respective complex medium.

### Media

For *P. putida* and *C. necator strains* LB medium (10 g·L^− 1^ tryptone, 5 g·L^− 1^ yeast extract, 5 g·L^− 1^ sodium chloride) was used. NB medium as suggested by DSMZ (5 g·L^− 1^ peptone, 3 g·L^− 1^ meat extract) was used for *A. australica*. Halobacter medium 372 from DSMZ (5 g·L^− 1^ yeast extract, 5 g·L^− 1^ casamino acids, 1 g·L^− 1^ sodium glutamate, 2 g·L^− 1^ KCl, 3 g·L^− 1^ sodium citrate, 20 g·L^− 1^ MgSO_4_ × 7H_2_O, 200 g·L^− 1^ NaCl, 36 m g·L^− 1^ FeCl_2_ × 4H_2_O, 0.36 mg·L^− 1^ MnCl_2_ × 4H_2_O) has been used as complex medium for *H. mediterranei*. For agar plates, 15 g·L^− 1^ agar has been added.

A modified Schlegel medium (4.5 g·L^− 1^ Na_2_HPO_4_, 1.5 g·L^− 1^ KH_2_PO_4_, 4.4 g·L^− 1^ (NH4)_2_SO_4_, 20.9 g·L^− 1^ MOPS buffer, 0.05 g·L^− 1^ ammonium ferric citrate, 0.01 g·L^− 1^ CaCl_2_ × 2H_2_O, 0.2 g·L^− 1^ MgSO_4_ × 7H_2_O, 1 mL/L SL6 trace element solution) was used as a mineral salt medium for cultivation of *P. putida* and *C. necator* [15]. SL6 trace element solution consisted of 1 g·L^− 1^ ZnSO_4_ × 7H_2_O, 0.3 g·L^− 1^ MnCl_2_ × 4H_2_O, 3 g·L^− 1^ H_3_BO_3_, 2 g·L^− 1^ CoCl_2_ × 6H_2_O, 0.1 g·L^− 1^ CuCl_2_ × 2H_2_O, 0.2 g·L^− 1^ NiCl_2_ × 6H_2_O, 0.3 g·L^− 1^ Na_2_MoO_4_ × 2 H_2_O. pH was adjusted to 7.2 with 4 M KOH.

The mineral salt medium used for *A. australica* was modified from Gahlawat et al. (4,27 g·L^− 1^ NH_4_Cl, 0.2 g·L^− 1^ MgSO_4_, 3.25 g·L^− 1^ KH_2_PO_4_, 3.25 g·L^− 1^ Na_2_HPO_4_, 20.9 g·L^− 1^ MOPS buffer and 1.5 mL/L TES trace element solution). TES consisted of 6 g·L^− 1^ ammonium ferric citrate, 0.3 g·L^− 1^ CaCl_2_ × 2H_2_O, 0.3 g·L^− 1^ H_3_BO_3_, 0.2 CoCl_2_ × 6H_2_O, 0.1 ZnSO_4_ × 7H_2_O, 0.03 g·L^− 1^ MnCl_2_ × 4H_2_O, 0.03 Na_2_MoO_4_ × 2H_2_O, 0.02 g·L^− 1^ NiSO_4_ × 7H_2_O and 0.01 g·L^− 1^ CuSO_4_ × 5H_2_O. pH was adjusted to 7.0 with 4 M NaOH [16]. 

For *H. mediterranei* mineral salt medium was adopted from Wang and Zhang 2021 (156 g·L^− 1^ NaCl, 4.4 g·L^− 1^ NH_4_Cl, 13 g·L^− 1^ MgCl_2_ × 6H_2_O, 20 g·L^− 1^ MgSO_4_ × 7H_2_O, 0.67 g·L^− 1^ CaCl_2_ × 2H_2_O, 4 g·L^− 1^ KCl, 0.5 g·L^− 1^ NaBr, 0.006 g·L^− 1^ ferric ammonium citrate, 0.5 g·L^− 1^ KH_2_PO_4_, 20.9 g·L^− 1^ MOPS, 0.001 g·L^− 1^ and 5 mL/L trace element solution). Trace element solution consisted of ZnSO_4_ × 7H_2_O, MnCl_2_ × 4H_2_O, 0.003 g·L^− 1^ H_3_BO_3_, 0.002 g·L^− 1^ CoCl_2_ × 6H_2_O, 0.0001 g·L^− 1^ CuCl_2_ × 2H_2_O, 0.0002 g·L^− 1^ NiCl2 × 6H2O, and 0.0003 g·L^− 1^ Na_2_MoO_4_. pH was adjusted to 7.0 with 4 M NaOH [17]. 

### Growth experiments

For preculture preparation, single colony from agar plates have been aseptically transferred to a 50 mL falcon tube, filled with a 5 mL complex medium. *P. putida*, *C. necator* and *A. australica* were cultivated at 30 °C, *H. mediterranei* at 37 °C. The rotary shaker (Infors HT, Switzerland) was set to 200 rpm. Once the culture medium reached at least OD_600_ 0.7 the tube was aseptically transferred to a 500 mL baffled shake flask with 50 mL complex medium and incubated at 120 rpm. Once the preculture reached OD_600_ of at least 1, the 48 wells BioLector^®^ flowerplates (Beckmann Coulter, USA) were filled with 950 µL of mineral salt medium containing carboxylic acids and inoculated with preculture broth to reach an OD_600_ of 0.1 (except *A. australica* at OD_600_ of 0.05), distilled water was used to fill the volume to 1000 µL. The carboxylic acid tested in this study were formic acid, propionic acid, butyric acid, valeric acid and levulinic acid, and acetic acid (The concentrations of the tested carboxylic acids are presented in the supplementary information Appendix I). The plates were incubated at temperatures specific to each microorganism at 1100 rpm in the BioLector^®^ system (Beckmann Coulter, USA) which equals to an OTR of roughly 50 mmol·L^− 1^·h^− 1^. Parameters such as pH, pO_2_ and backscatter (λ_Ext_ = 660 nm) were automatically monitored and recorded every 10 min by the system. To prevent evaporation while allowing air transfer, gas-permeable sealing foils were used and humidity was set to 85%. All experiments have been performed in biological triplicate.

### Determination of specific growth rate and duration of lag phase

**Fig. 1:**
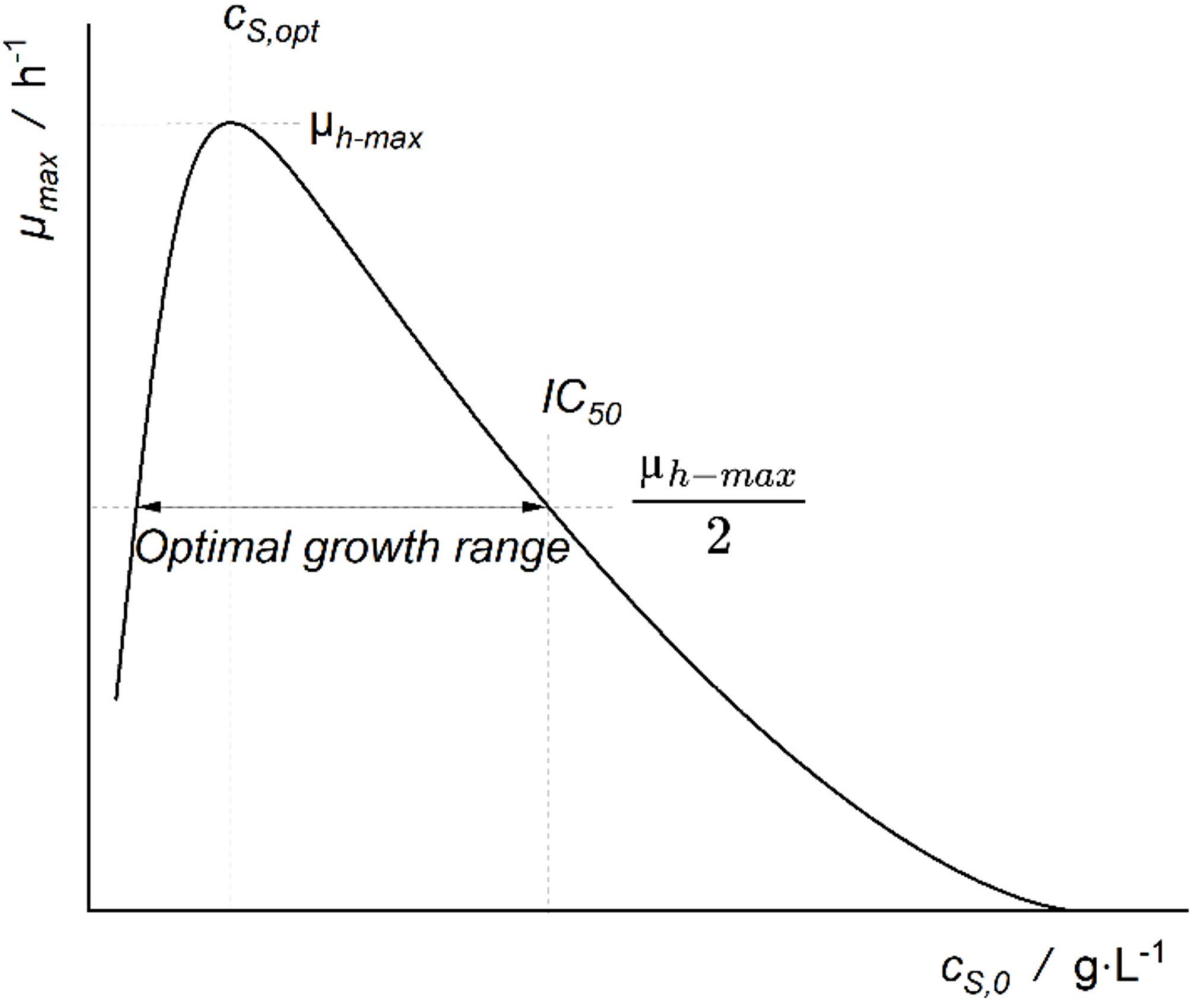
Han-Levenspiel Inhibitory kinetic model and the various parameters used in this study

The maximum specific growth rate (*µ*_*max*_) has been determined by plotting the natural logarithm of backscatter values against cultivation time. Linear regression was applied during the exponential phase of which the slope is equivalent to *µ*_*max*_ [18]. In the case of biphasic growth behaviour, the first growth phase has been chosen for the calculation of *µ*_*max*_ to guarantee initial substrate concentrations. The specific growth rates were plotted against the initial substrate concentrations and Han-Levenspiel inhibitory kinetics were fitted to the data. From the fitted Han-Levenspiel models (Fig. 1) the inhibitory concentration (IC_50_), the highest determined specific growth rate (*µ*_*h−max*_) and the optimal growth or tolerance range (concentration at which *µ*_*max*_ > 50% *µ*_*h−max*_) for each acid was estimated. IC_50_ was calculated as the concentration at which *µ*_*max*_ is inhibited by *50%* o *µ*_*h−max*_ and it offers therefore a measure of the toxicity of a substrate onto a microorganism. Statistical errors have been determined as standard errors and propagated by Gaussian error propagation. Statistical significance has been determined by t-test. Duration of the lag phase (λ) has been calculated according to Baranyi and Pin et al. 1999 [18].

The backscatter was used for the measurement of total biomass, which cannot differentiate between active biomass and PHA production in the biomass. As *C. necator*, *P. putida* and *H. mediterranei* require limiting conditions (N limitation) to induce PHA production, to prevent this limitation the medium was supplied with enough nitrogen during the cultivation. However, *A. australica* on the other hand is known to show growth-associated PHA production on sugars. Therefore, in the case of *A. australica*, the backscatter signals might contain some PHA along with the biomass.

## Results and discussion

The growth characteristics of the four microorganisms—*C. necator* H16, *P. putida* KT2440, *A. australica* DSM 1124, and *H. mediterranei* DSM 1411— are evaluated in each carboxylic acid in the following sections. Alle the raw data from the growth experiments are presented in the supplementary material (Appendix I).

### Formic acid as a carbon source

**Fig. 2:**
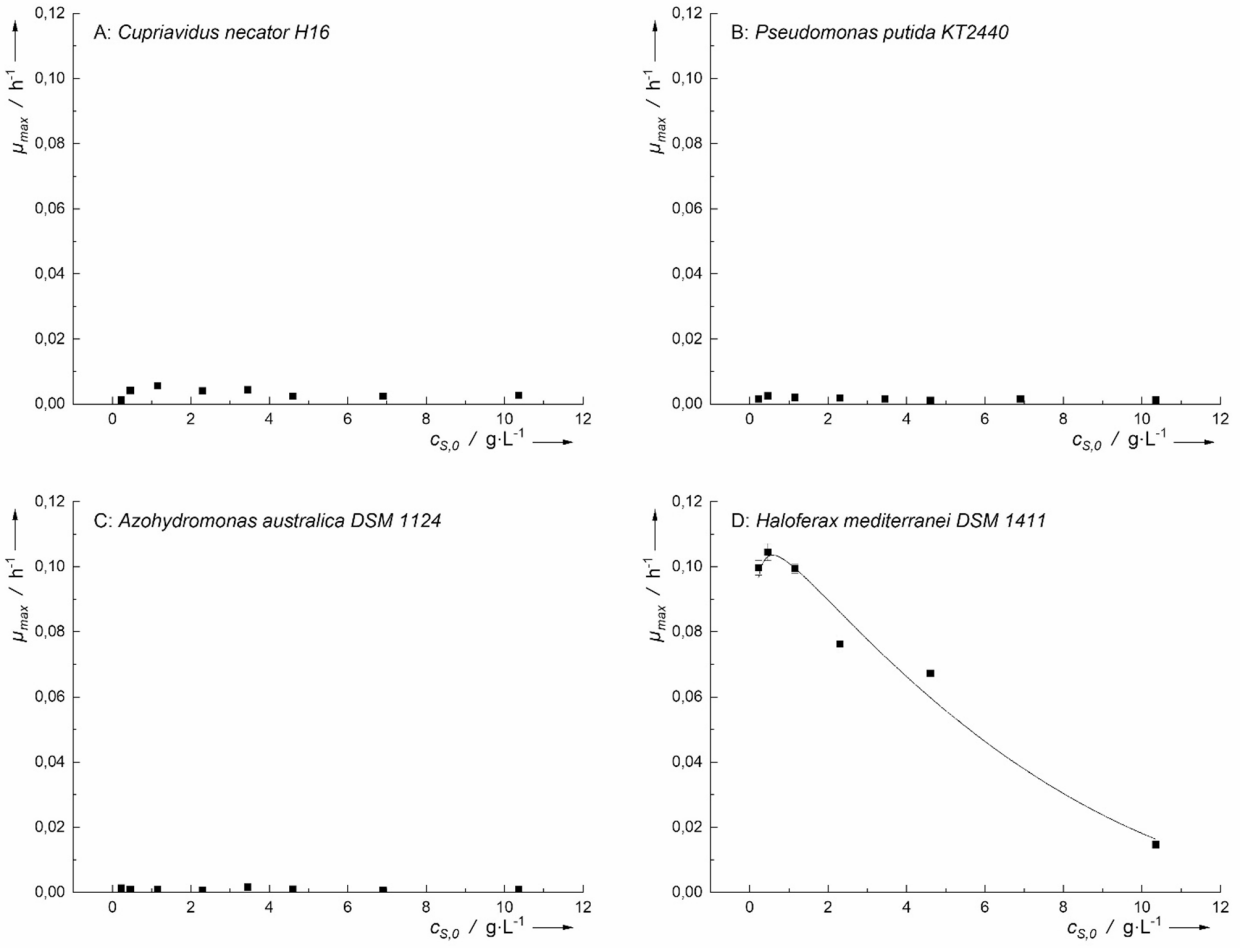
Maximum specific growth rates (*µ*_*max*_) of* C. necator*, *P. putida*, *A. australica* and *H. mediterranei* on formic acid. Inhibitory growth kinetic according to Han-Levenspiel was fitted only for *H. mediterranei*

The four microorganisms were cultivated in media containing varying concentrations of formic acid as the sole carbon and energy source. Among the tested microorganisms, only *H. mediterranei* exhibited growth at formic acid concentrations up to 10.4 g·L⁻¹, while the other three bacteria showed no significant growth (Fig. 2).

The highest specific growth rate (*µ*_*h−max*_) for *H. mediterranei* was 0.104 ± 0.003 h^− 1^ at *c*_*S,0*_ of 0.5 g·L^− 1^. However, at *c*_*S,0*_ of 0.23 g·L^− 1^, a *µ*_*max*_ of 0.100 ± 0.003 h^− 1^ was observed, which was not significantly (*p* > 0.05) lower. This implies that optimal *µ*_*max*_ could be at *c*_*S,0*_ < 0.5 g·L^− 1^. Looking at the inhibitory concentration of formic acid, which was determined by *IC*_*50*_, for this organism was observed to be 5.6 g·L^− 1^, as observed in Fig. 2D.

**Fig. 3:**
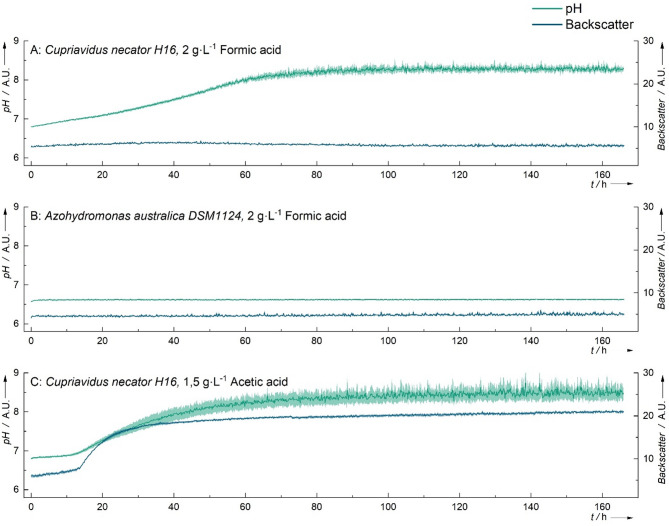
Backscatter and pH course of *C. necator* on formic acid and acetic acid and *A. australica* on formic acid, *n* = 3

An interesting phenomenon was observed when *C. necator* was cultivated in formic acid. Despite the absence of growth (indicated by no change in backscatter), the pH increased during fermentation (Fig. 3A). In contrast, this pH shift was not observed in *P. putida* or *A. australica*, where the pH remained stable throughout cultivation (see Fig. 3B). Notably, a similar trend in pH change was observed during *C. necator*’s growth in acetic acid (Fig. 3C). These findings suggest that *C. necator* H16 was able to metabolize formic acid, as indicated by the pH shift, whereas *P. putida* and *A. australica* did not exhibit metabolic activity.

The growth of *C. necator* in formic acid varies across publications. While some studies report higher growth rates up to 0.18 h^− 1^_,_ others report growth with low *µ*_*max*_ (doubling time of 5 d) [19–21] or no growth [9]. This discrepancy can be attributed to *C. necator*’s formic acid metabolism, which relies on two independent formate dehydrogenases, at least one of which is influenced by the cell’s redox state [19].

A reason for the low growth of *C. necator* could be the usage of Calvin-Benson-Bassham-cycle (CBB) for the metabolism of formic acid. This metabolic pathway takes 7 moles ATP for the production of one pyruvate from formic acid [22], while only 2.5 mol ATP could be produced per mole formic acid [23]. In contrast, the genomic data suggest that a *Haloferax* strain has the ability to partially fixate formic acid directly into acetyl-CoA using the formate tetrahydrofolate cyclo-ligase enzyme through a complex metabolic pathway [24, 25]. This formic acid metabolic pathway appears to be more efficient, leading to better growth in *H. mediterranei* compared to *C. necator*.

Variations in process parameters and preculturing methods may affect the redox state, thereby influencing growth and leading to contradictory findings on *C. necator*’s ability to grow on formate [19].

### Effect of acetic acid as a carbon source on microbial growth

**Fig. 4:**
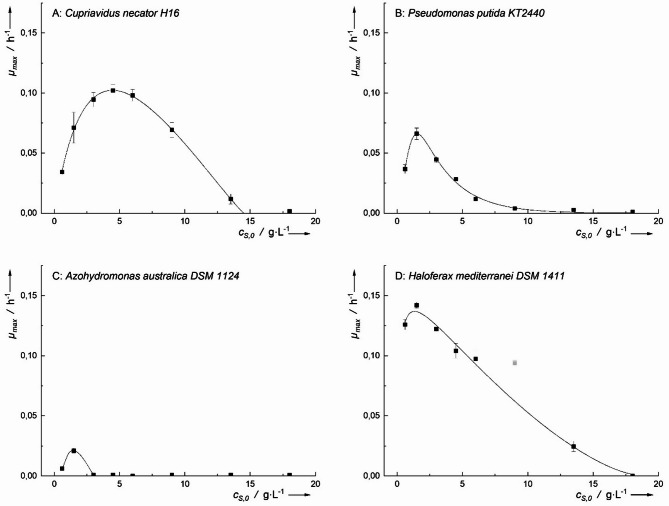
Maximum growth rates plotted against the initial concentration of acetic acid for various PHA-producing microorganisms. Inhibitory according to Han-Levenspiel have been fitted to the data. Points not considered for the calculation have been greyed

Microbial growth was observed in all tested microorganisms when cultivated with acetic acid as the sole carbon source (Fig. 4). The highest specific growth rates (*µ*_*h−max*_) were recorded at an acetic acid concentration of 1.5 g·L⁻¹ for three microorganisms: *H. mediterranei* (0.142 ± 0.002 h⁻¹), *P. putida* (0.066 ± 0.005 h⁻¹), and *A. australica* (0.021 ± 0.002 h⁻¹). In constrast, *C. necator* reached its *µ*_*h−max*_ (0.102 ± 0.005 h⁻¹) at a higher concentration of 4.5 g·L⁻¹.

The *µ*_*h−max*_ of *H. mediterranei* was significantly higher than that of *C. necator* (*p* < 0.05) and strongly significantly higher than that of *P. putida* and *A. australica* (*p* < 0.01). Among all tested microorganisms, *A. australica* exhibited the lowest *µ*_*h−max*_ on acetic acid, which may be attributed to an impaired TCA cycle, as reported for the closely related *Azohydromonas lata* DSM 1123 [12].

As shown in Fig. 4A, the optimal growth range (*µ*_*max*_ > 50% *µ*_*h−max*_, determined using Han-Levenspiel kinetics) for *C. necator* was between 0.95 g·L⁻¹ and 10.5 g·L⁻¹ acetic acid. Compared to the other microorganisms, *C. necator* exhibited a broader optimal growth range and a higher inhibitory concentration (*IC*_*50*_ = 10.5 g·L⁻¹), followed by *H. mediterranei * (*IC*_*50*_ = 8.2 g·L⁻¹), *P. putida* (IC₅₀ = 3.8 g·L⁻¹), and A. *australica* (IC₅₀ = 2.5 g·L⁻¹) in acetic acid.

These findings suggest that *C. necator* and *H. mediterranei* are the most suitable candidates for growth when acetic acid is used as a substrate.

### Microbial growth on butyric acid

**Fig. 5:**
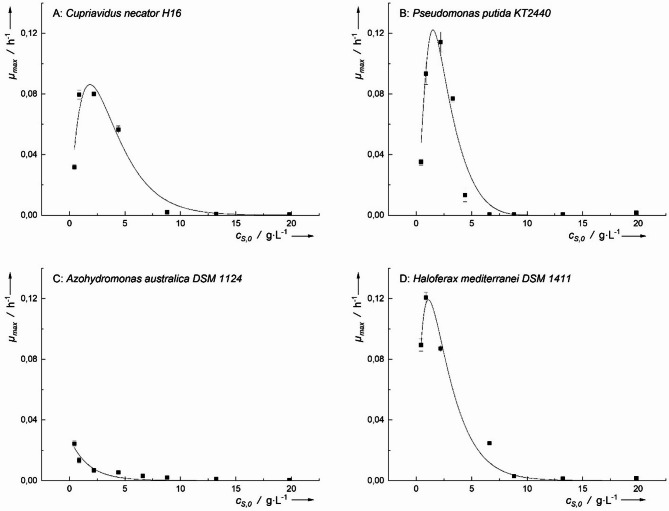
Maximum growth rates plotted against the initial concentration of butyric acid for various PHA-producing microorganisms. Inhibitory according to Han-Levenspiel have been fitted to the data

The suitability of butyric acid as a carbon source for growth of all four microorganisms was evaluated, and the respective growth kinetics are presented in Fig. 5. The highest *µ*_*max*_ was observed for *H. mediterranei* (*µ*_*max*_ = 0.121 ± 0.004 h^− 1^ at 0.9 g·L^− 1^) and *P. putida* (*µ*_*max*_ = 0.114 ± 0.007 h^− 1^ at 2.2 g·L^− 1^). However, the t-test revealed that the *µ*_*h−max*_ of *H. mediterranei* was not significantly higher (*p* > 0.05) compared to *P. putida*. In the case of *C. necator*, a *µ*_*h−max*_ of 0.080 ± 0.001 h^− 1^ was observed at 2.2 g·L^− 1^, which was significantly lower than the growth rates of *H. mediterranei* or *P. putida*.

Compared to acetic and formic acid, *H. mediterranei* exhibited a lower inhibitory concentration (*IC₅₀* = 3.3 g·L⁻¹), indicating greater sensitivity to butyric acid. Similarly, *P. putida* had a lower *IC₅₀* (3.6 g·L⁻¹) than in acetic acid, suggesting that butyric acid exerted a stronger inhibitory effect. Although *C. necator* showed a lower growth rate in butyric acid compared to other microorganisms, the inhibitory concentration (*IC*_*50*_ of 5.2 g·L^− 1^) was comparatively higher. This indicates that *C. necator* is more tolerant to butyric acid.

Compared to acetic and formic acid, the growth of *H. mediterranei* exhibited a lower inhibitory concentration (*IC*_*50*_ *=* 3.3 g·L^− 1^), indicating greater sensitivity to butyric acid. Analogue to *H. mediterranei*, the inhibitory concentration in *P. putida* was also lower (*IC*_*50*_ *=* 3.6 g·L^− 1^) compared to acetic acid, indicating that butyric acid has a stronger inhibitory effect than acetic acid. Although *C. necator* showed lower *µ*_*h−max*_ in butyric acid compared to *H. mediterranei* or *P. putida*, the inhibitory concentration (IC_50_ of 5.2 g·L^− 1^) was comparatively higher. This indicates that *C. necator* is more tolerant to butyric acid.

In contrast, *A. australica* exhibited a low specific growth rate on butyric acid (*µ*_*max*_ = 0.024 ± 0.002 h⁻¹ at 0.4 g·L⁻¹). Notably, the growth rate increased as the butyric acid concentration decreased (Fig. 5C). This suggests that further reducing the concentration below 0.4 g·L⁻¹ might enhance growth. However, from a practical standpoint, maintaining such low substrate concentrations in a bioreactor is not feasible. In pilot- or industrial-scale bioreactors, where highly concentrated feeds are typically used, achieving a homogeneous distribution of butyric acid at low concentrations is challenging. Due to longer mixing times in larger-scale systems, precise control over substrate concentration at every location within the bioreactor becomes increasingly difficult.

### Growth behavior of microorganisms on 3-HV precursors: propionic, valeric, and levulinic acids

Poly(3-hydroxybutyrate-co-3-hydroxyvalerate) (PHBV) is a copolymer of PHA that exhibits enhanced properties compared to PHB. While PHB is brittle and has poor processability, the incorporation of 3-hydroxyvalerate (3-HV) units into the polymer chain improves flexibility, elasticity, and thermal properties [26–28]. This makes PHBV more suitable for applications such as biodegradable packaging and coating [29, 30]. The ratio of 3-hydroxybutyrate (3-HB) to 3-hydroxyvalerate (3-HV) influences the mechanical properties, with higher HV content generally leading to greater flexibility and lower crystallinity.

Propionic acid, valeric acid, and levulinic acid are direct precursors to 3-hydroxyvalerate (3-HV) [31], a monomer in the copolymer PHBV. These acids enable microorganisms like *C. necator* to produce PHBV, yet studies on microbial growth behaviours using these acids remain scarce. This section discusses the growth characteristics of microorganisms in these acids.

### Growth on propionic acid

**Fig. 6:**
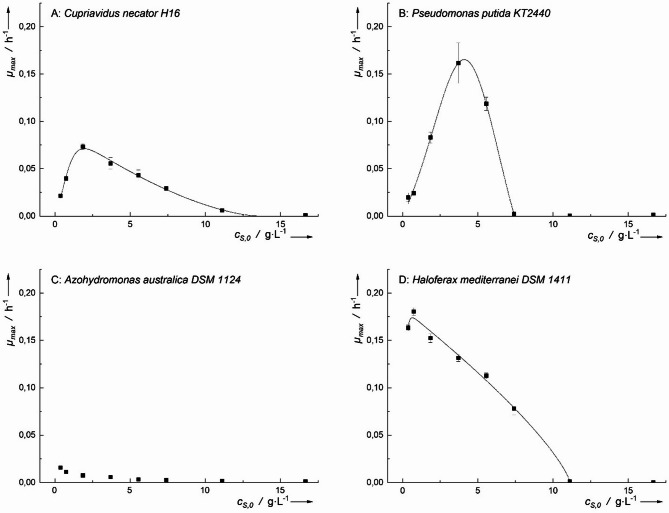
Maximum growth rates plotted against the initial concentration of propionic acid for various PHA-producing microorganisms. Inhibitory according to Han-Levenspiel have been fitted to the data

Propionic acid exhibited varying effects on microbial growth (Fig. 6). *P. putida* and *Haloferax mediterranei* achieved the highest *µ*_*max*_ of 0.16 ± 0.02 h⁻¹ at 3.7 g·L⁻¹ and 0.18 ± 0.005 h⁻¹ at 0.7 g·L⁻¹. These growth rates were significantly higher compared to those observed with acetic acid (*p* < 0.05 and *p* < 0.01, respectively). In contrast, *C. necator* had a significantly lower growth rate (*µ*_*max*_ = 0.073 ± 0.003 h⁻¹ at 1.9 g·L⁻¹), and *Azohydromonas australica* showed no detectable growth on propionic acid.

Toxicity assessments indicated that *C. necator* and *H. mediterranei* exhibited similar tolerance levels to propionic acid, with an inhibitory concentration (*IC₅₀*) of 6.7 g·L⁻¹. *P. putida*, however, demonstrated a lower tolerance, with an *IC₅₀* of 3.8 g·L⁻¹ (Fig. 6).

### Growth on valeric acid

Valeric acid proved to be the most toxic among the three precursor acids. *C. necator* and *H. mediterranei* showed the highest growth rates (*µ*_*max*_ = 0.093 ± 0.003 h⁻¹ and 0.09 ± 0.02 h⁻¹, respectively) at 1 g·L⁻¹. *P. putida* exhibited a slower growth rate (0.077 ± 0.003 h⁻¹) at a higher concentration of 3.9 g·L⁻¹), while no growth of A. *australica* was observed.

**Fig. 7:**
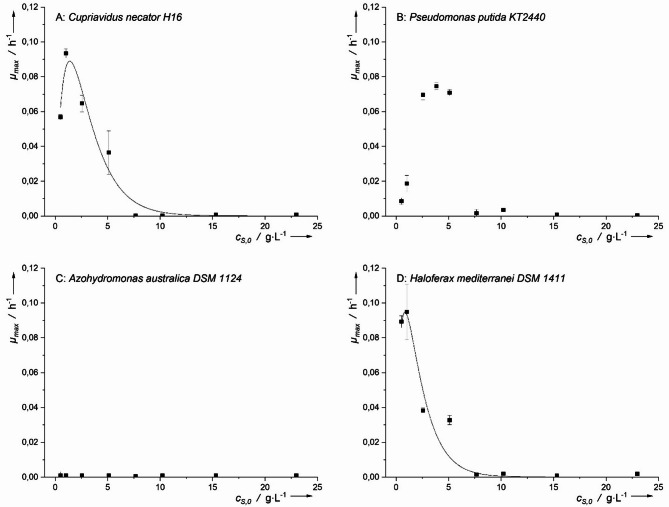
Maximum growth rates plotted against the initial concentration of valeric acid for various PHA-producing microorganisms. Inhibitory according to Han-Levenspiel have been fitted to the data

Valeric acid inhibited growth at lower concentrations compared to other carboxylic acids. The inhibitory concentration (*IC₅₀*) was 3.9 g·L⁻¹ for *C. necator* and 2.7 g·L⁻¹ for *H. mediterranei*. Although no suitable Han-Levenspiel model could be fitted for *P. putida*, concentrations above 5 g·L⁻¹ were clearly inhibitory, as *µ*_*max*_ dropped to nearly zero beyond this threshold (Fig. 7).

### Growth on levulinic acid

**Fig. 8:**
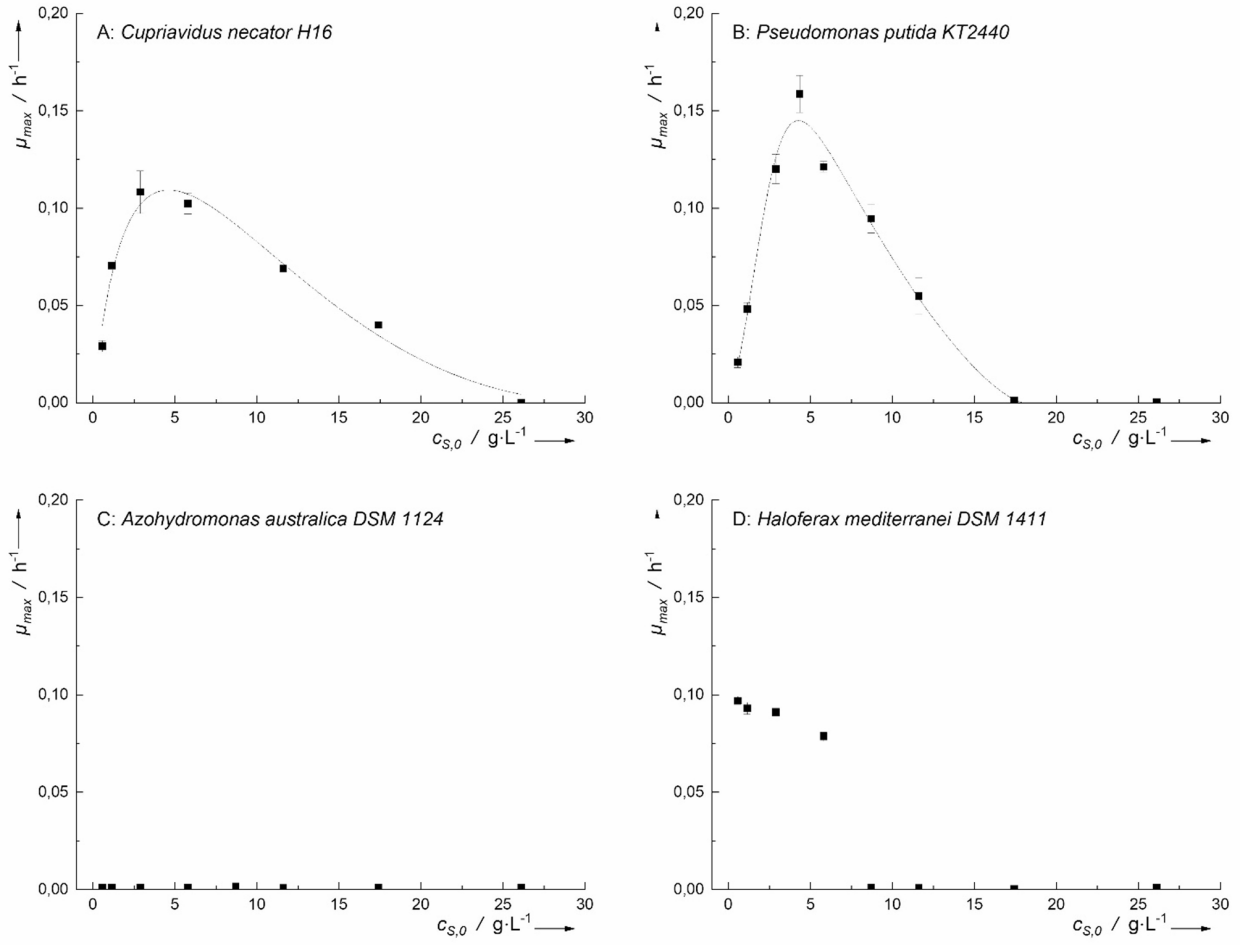
Maximum growth rates plotted against the initial concentration of levulinic acid for various PHA-producing microorganisms. Inhibitory according to Han-Levenspiel have been fitted to the data. Points not considered for the calculation have been greyed

Levulinic acid was less toxic for *C. necator* and *P. putida* than valeric acid due to their higher *IC*_*50*_ on Levulinic acid. *P. putida* showed the highest growth rate (*µ*_*h−max*_ = 0.16 ± 0.01 h⁻¹ at 3 g·L⁻¹). Optimal growth for *P. putida* occurred within the range of 1.8 to 9.6 g·L⁻¹ levulinic acid, whereas valeric acid was completely inhibitory at 7.7 g·L⁻¹ (Fig. 8).

*C. necator* demonstrated a *µ*_*h−max*_ of 0.11 ± 0.01 h⁻¹ at 3 g·L⁻¹ and had a much broader optimum growth concentration range (0.9–14.0 g·L⁻¹) compared to valeric and propionic acids. In fact, its inhibitory concentration on levulinic acid was three times higher than on valeric acid. *H. mediterranei* exhibited its highest growth rate (*µ*_*max*_ = 0.097 ± 0.002 h⁻¹ at 0.6 g·L⁻¹), although its growth data did not fit well with the Han-Levenspiel model.

Among the three tested 3-HV precursor acids, levulinic acid demonstrated the most favorable kinetic profile for *C. necator*, which had higher *IC*_*50*_ compared to propionic and valeric acids. *H. mediterranei* also showed promising growth on levulinic acid, although the toxicity data did not fit the model sufficiently. Valeric acid was consistently the most toxic precursor, particularly for *C. necator* and *H. mediterranei*. From a kinetic perspective, propionic and levulinic acids appear to be the most suitable 3-HV precursor substrates for microbial growth.

### Correlation between hydrophobicity and toxicity of carboxylic acids

The link between toxicity and hydrophobicity is well-documented and has been observed in many substances, such as alcohol [32–34]. This study also observed a general correlation between the hydrophobicity of carboxylic acids and their toxicity (as measured by the *IC₅₀*).

**Fig. 9:**
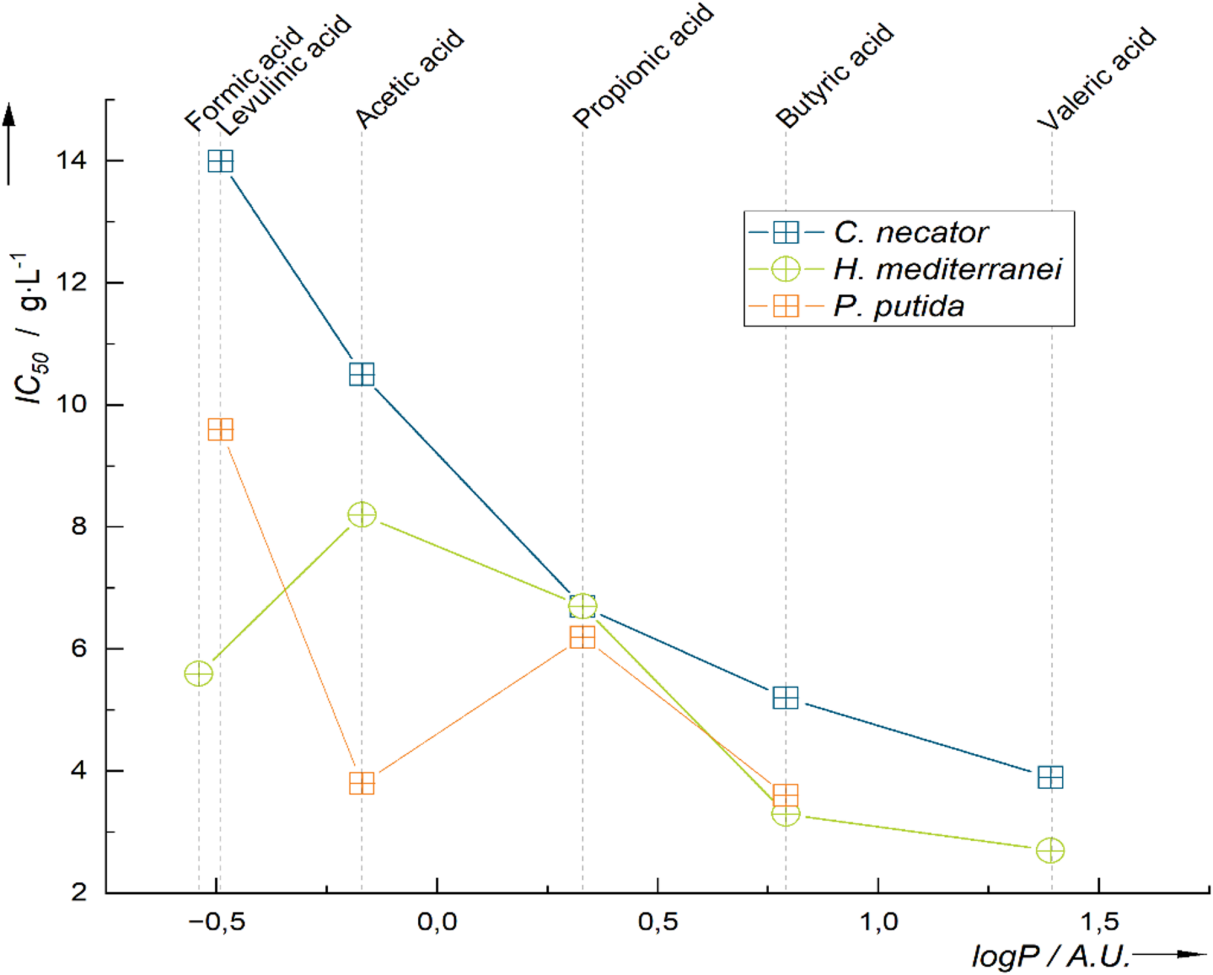
IC_50_ plotted against the decadic logarithm of the octanol-water partition coefficient for PHA producers

The decadic logarithm partition coefficient (*logP*) of substances in water and octanol generally quantifies the hydrophobicity of a substance. For the carboxylic acids, *logP* values were obtained from the publicly available DrugBank database [35]. A more hydrophilic compound typically has a lower *logP* value (< 0), while hydrophobic compounds have a higher *logP* value (> 0) (Fig. 9).

For *C. necator*,* P. putida* and *H. mediterranei*, IC_50_ values were plotted against the *log P* values of each carboxylic acid, as shown in Fig. 9. *A. australica* was not included because no growth was observed. It was observed that growth was generally more inhibited when the microorganisms were grown on more hydrophobic substrates. However, formic acid did not follow this trend—it exhibited higher toxicity despite having the lowest *logP* value (-0.54). In contrast, levulinic acid, with a slightly higher *logP* value of -0.49, showed the lowest toxicity, as expected. The mechanism behind the toxicity of formic acid cannot be attributed to its hydrophobicity. Instead, formic acid inhibits aromatic amino acid biosynthesis in *C. necator* [36] thus inhibiting the growth. In *P. putida* the same trend is visible with the exception of acetic acid, which was more inhibited than propionic acid, although the hydrophobicity would suggest otherwise.

In summary, the lower hydrophobicity of levulinic acid makes it a less toxic alternative to valeric and propionic acids for PHBV production in *C. necator and thus a preferred substrate*. This could potentially lead to more robust production processes in the future.

### Effect of carboxylic acids on the lag phase

**Fig. 10:**
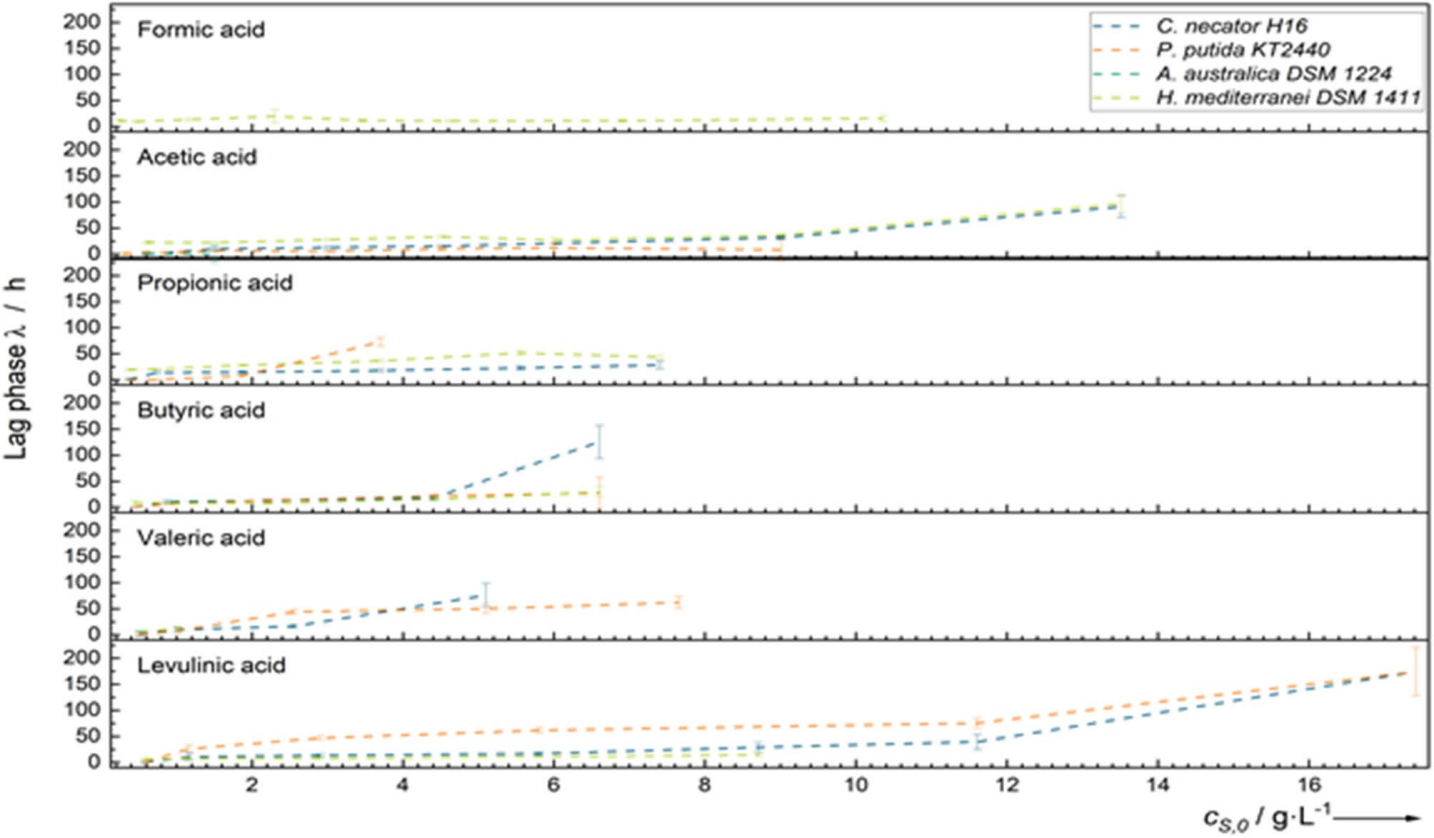
Lag Phases of *C. necator*, *P. putida*, *A. australica*, and *H. mediterranei* on various carboxylic acids

When determining the inhibitory concentration (*IC*_*50*_) of each carboxylic acid for different microorganisms, our study also revealed that high carboxylic acid concentrations led to significantly longer lag phases, as shown in Fig. 10. This effect is especially pronounced if the microorganisms are grown in carboxylic acid concentrations far above the optimum (*c*_*S,0*_ > > *c*_*S, opt*_). For example, *C. necator* showed a lag phase of 12 ± 2 h at 2.2 g·L^− 1^ butyric acid (*c*_*S, opt*_ = 1.9 g·L^− 1^). At a concentration of 6.6 g·L^− 1^ butyric acid, we even observed a lag phase of 126 ± 32 h.

From a bioprocessing perspective, shorter lag phases are preferred because longer lag phases extend the total fermentation time, directly affecting operational costs. Therefore, selecting an appropriate carboxylic acid concentration is crucial to balancing higher growth rates with an optimal lag phase for efficient bioreactor operation.

As discussed in the previous section, *C. necator* exhibited an IC₅₀ of 14 g·L^− 1^ for levulinic acid (Table 1). However, at this concentration, the lag phase is expected to strongly increase (40 ± 15 h at 11.61 g·L^− 1^) making it impractical for industrial applications despite its tolerance. Thus, higher carboxylic acid concentrations might support growth, they also lead to extended lag phases, making it crucial to find an optimal balance for industrial bioreactor.

In some cases, lag phase duration can be minimized using an optimized preculture cascade. This approach involves gradually adapting the microbial culture to increasing concentrations of carboxylic acids before transferring them to the main bioreactor. This pre-adaptation helps microorganisms to acclimatize themselves in advance, thereby reducing lag phase and improving overall bioprocess efficiency.

### General discussion on microorganisms

#### Azohydromonas australica

As discussed in the previous section, *A. australica* DSM 1124 showed no growth on most of the carboxylic acids tested in this study. This observation is consistent with the findings of Chen et al., who also reported no growth of *A. australica* on propionic or valeric acid [13]. In contrast, Haage et al. reported a growth rate of *µ* = 0.13 h⁻¹ for *A. australica* on valeric acid as the sole carbon source [37]. This discrepancy may be due to the strain maintenance method used by Haage et al., where single colonies were transferred to fresh plates every five weeks, a process that carries a higher risk of contamination and mutation.

Despite the lack of growth observed, carboxylic acids could still serve as substrates for PHA production. Both Chen et al. and Haage et al. reported that *A. australica* can produce 3-hydroxyvalerate (3-HV) in the polymer when 3-HV precursors are added to the medium. This suggests that, although there is no consensus regarding the growth of *A. australica* on carboxylic acids, it is agreed in the literature that PHA production is feasible from these substrates. Notably, the successful production of PHBV by *A. australica* was only achieved when both propionic and acetic acids were supplied together in the medium [13].

Carboxylic acids, are typically metabolized directly or via β-oxidation pathways, which feed into the TCA or similar methyl citrate cycle for growth and maintenance. For the closely related *A. lata* DSM1123, an impaired TCA cycle has been reported, which led to growth-related PHB production due to an overflow metabolism of acetyl-CoA [12]. Given that *A. australica* DSM 1124 is also known for growth-associated PHA production [38], it is plausible that a similar impaired TCA metabolism may be the reason for very slow or no significant growth of *A. australica* on carboxylic acids in our experiments.

#### Pseudomonas putida

Our study demonstrated that *Pseudomonas putida* was capable of growth on most of the carboxylic acids tested, except for formic acid. This is consistent with previous findings by researchers who studied the growth of *P. putida* on acids such as acetate [39], butyrate [8, 40] or levulinate [41]. The inability of *P. putida* to metabolize formic acid, as observed in our experiments, is in agreement with Turlin et al., who suggested that the metabolic pathway for C1 substrates like formic acid must be incorporated into the metabolism of *P. putida* through genetic engineering [42].

When comparing the specific growth rates calculated in our study to those published in other works, we observed that growth rates in the microbioreactor system were lower than those observed in bioreactor or shake flask studies. For example, Cerrone et al. [40] reported a maximum specific growth rate of 0.30 h⁻¹ in a fed-batch bioreactor using *P. putida* CA-3 in butyric acid, while our study found a much lower growth rate of 0.114 ± 0.007 h⁻¹. In case of growth studies of *P. putida* in microbioreactor system (96-well plates), Escapa et al. [39] reported a growth rate of 0.024 h⁻¹ on acetic acid, which is significantly lower than the growth rate of 0.066 ± 0.005 (*p* > 0.01) observed in our study.

#### Cupriavidus necator

*C. necator* H16 also exhibited growth on all tested carboxylic acids, except formic acid. Previous studies have shown similar findings [43–47], but no systematic studies have thoroughly investigated the inhibitory growth behavior of *C. necator* on these substrates. Vu et al. performed bioreactor studies on *C. necator* DSM 545, where they observed growth on 5 g·L⁻¹ of acetate, propionate, and butyrate, which is consistent with our results. Notably, our results differ in terms of valeric acid tolerance: while Vu et al. observed no growth at 5 g·L⁻¹ of valeric acid, our study showed growth at this concentration, likely due to differences in the strains of *C. necator* used [48]. 

Additionally, Jawed et al. conducted microbioreactor studies on *C. necator* H16 with carboxylic acids in a narrower concentration range (≤ 50 mM) [9]. Although their study did not report specific growth rates or inhibitory kinetics, they found that growth was strongly inhibited at 50 mM (approximately 4.4 g·L⁻¹) of butyrate, which is consistent with our results (Table 1). However, for valeric acid, we found growth even at ≥ 3.5 g·L⁻¹, while Jawed et al. reported the opposite. The growth of *C. necator* H16 at valeric acid concentration ≥ 3.5 g·L⁻¹ was accompanied by a long lag phase of 73 ± 7 h in our experiments (Fig. 10). This delay in growth might explain the discrepancies with the findings of Jawed et al. and Vu et al., who may have terminated their experiments too early before observing growth due to the lag phase.

#### Haloferax mediterranei

*H. mediterranei* was cultivated very successfully on carboxylic acids. Previous studies by Ferre-Guell and Winterburn [49] and Gonzalez and Winterburn [50] showed that *H. mediterranei* can grow on short-chain carboxylic acid at concentrations higher than 100 mM. However, in our study, we found optimal growth only with acetic and propionic acid. The growth curves published by these authors on butyric and valeric acid showed very low growth rates, as they were cultivated in a concentration far above the optimal concentration. Specific growth rates might be improved by using lower substrate concentrations, as demonstrated in this study.

Overall, our findings suggest that most carboxylic acids can serve as suitable substrates for microbial growth, particularly for *P. putida*, *C. necator*, and *H. mediterranei*. However, the specific growth rate and tolerance to each substrate vary, and some carboxylic acids, like formic and valeric acid, exhibit stronger inhibitory effects on microbial growth, thus requiring a precise feed control in the bioreactor.

**Table 1:** Summary of the growth kinetics from the Han-levenspiel inhibitory growth model

Microorganisms	Formic acid			Acetic acid			Propionic acid			Butyric acid			Valeric acid			Levulinic acid		
	*µ*_*h−max*_ / h^− 1^	c_opt_ / g·L^− 1^	IC_50_ / g·L^− 1^	*µ*_*h−max*_ / h^− 1^	c_opt_ / g·L^− 1^	IC_50_ / g·L^− 1^	*µ*_*h−max*_ / h^− 1^	c_opt_ / g·L^− 1^	IC_50_ / g·L^− 1^	*µ*_*h−max*_ / h^− 1^	c_opt_ / g·L^− 1^	IC_50_ / g·L^− 1^	*µ*_*h−max*_ / h^− 1^	c_opt_/ g·L^− 1^	IC_50_/ g·L^− 1^	*µ*_*h−max*_ / h^− 1^	c_opt_/ g·L^− 1^	IC_50_/ g·L^− 1^
*C. necator* **H16**	ng	ng	ng	0.102	4.4	10.5	0.072	1.9	6.7	0.086	1.9	5.2	0.089	1.5	3.9	0.109	4.6	14.0
*P. putida* **KT2440**	ng	ng	ng	0.066	1.5	8.2	0.165	4.1	3.8	0.122	1.5	6.2	mnf	mnf	3.6	0.145	4.3	9.6
*A. Australica* DSM 1124	ng	ng	ng	0.021	1.4	ng	ng	ng	ng	ng	ng	ng	ng	ng	ng	ng	ng	ng
*H. mediterranei* DSM 1411	0.103	0.6	5.6	0.137	1.3	8.2	0.174	0,6	6.7	0.119	1,1	3.3	0.095	0.08	2.7	mnf	mnf	mnf

*c*_*opt*_ Concentration at with highest specific growth rate (*µ*_*h−max*_) was observed. ng: No observable growth; mnf: Model not fitted, from Han-Levenspiel inhibitory growth model.

## Conclusion

Short-chain carboxylic acids are important intermediates for the production of polyhydroxyalkanoates (PHAs) by various microorganisms. This study provides comprehensive insights into the growth and inhibition kinetics of four key PHA-producing microorganisms, *Pseudomonas putida* KT2440, *Cupriavidus necator* H16, *Azohydromonas australica* DSM 1124, and *Haloferax mediterranei* DSM 1411, on a range of short-chain carboxylic acids.

The tested microorganisms exhibited distinct substrate preferences and tolerance levels. Acetic acid emerged as the most favorable substrate, enabling growth of all tested strains, including *A. australica*, which otherwise showed no growth on other acids. Notably, *C. necator* and *P. putida* demonstrated the broadest optimal growth range on acetic and levulinic acids, making these substrates particularly favorable for growth during PHA fermentation.

Formic acid, in contrast, only supported growth in *H. mediterranei*, highlighting its limited applicability for other strains. However, given its relevance as an intermediate in electrochemical CO₂ fixation, further research is warranted to optimize growth conditions for *C. necator* on formic acid.

*H. mediterranei* showed the ability to utilize all tested acids, but its requirement for high salinity media presents practical challenges for industrial application. *C. necator*, on the other hand, demonstrated a broader optimal growth range and higher inhibitory concentration, indicating a higher tolerance to carboxylic acids.

A significant correlation was observed between the hydrophobicity of the carboxylic acids and their toxicity — more hydrophobic acids tended to be more toxic. Additionally, higher acid concentrations led to prolonged lag phases, emphasizing the need for careful feed control and potential pre-adaptation strategies to improve fermentation performance.

In conclusion, this study underscores the importance of selecting substrate concentrations for process design, aiming to enhance microbial growth rates and overall bioprocess efficiency.

## Supplementary Information

Below is the link to the electronic supplementary material.

Supplementary Material 1.

## Data Availability

Data is provied within the manuscript or in the supplementary information files.
